# Association of Research and Development Investments With Treatment Costs for New Drugs Approved From 2009 to 2018

**DOI:** 10.1001/jamanetworkopen.2022.18623

**Published:** 2022-09-26

**Authors:** Olivier J. Wouters, Lucas A. Berenbrok, Meiqi He, Yihan Li, Inmaculada Hernandez

**Affiliations:** 1 Department of Health Policy, London School of Economics and Political Science, London, United Kingdom; 2 Department of Pharmacy and Therapeutics, University of Pittsburgh School of Pharmacy, Pittsburgh, Pennsylvania; 3 Division of Clinical Pharmacy, Skaggs School of Pharmacy and Pharmaceutical Sciences, University of California, San Diego, La Jolla

## Abstract

**Importance:**

Drug companies frequently claim that high prices are needed to recoup spending on research and development. If high research and development costs justified high drug prices, then an association between these 2 measures would be expected.

**Objective:**

To examine the association between treatment costs and research and development investments for new therapeutic agents approved by the US Food and Drug Administration (FDA) from 2009 to 2018.

**Design, Setting, and Participants:**

This cross-sectional study analyzed 60 drugs approved by the FDA between January 1, 2009, and December 31, 2018, for which data on research and development investments and list or net prices were available. Data sources included the FDA and SSR Health databases.

**Main Outcomes and Measures:**

The primary independent variable was estimated research and development investment. The outcome was standardized treatment costs (ie, annual treatment costs for both chronic and cycle drugs, and treatment costs for the maximum length of treatment recommended for acute drugs). Standardized treatment costs were estimated separately using list and net prices obtained from SSR Health at the time of launch and in 2021. To test the association between research and development investments and treatment costs, correlation coefficients were estimated and linear regression models were fitted that controlled for other factors that were associated with treatment costs, such as orphan status. Two models were used: a fully adjusted model that was adjusted for all variables in the data set associated with treatment costs and a parsimonious model in which highly correlated variables were excluded.

**Results:**

No correlation was observed between estimated research and development investments and log-adjusted treatment costs based on list prices at launch (*R* = −0.02 and *R*^2^ = 0.0005; *P* = .87) or net prices 1 year after launch (*R* = 0.08 and *R*^2^ = 0.007; *P* = .73). This result held when 2021 prices were used to estimate treatment costs. The linear regression models showed no association between estimated research and development investments and log-adjusted treatment costs at launch (β = 0.002 [95% CI, −0.02 to 0.02; *P* = .84] in the fully adjusted model; β = 0.01 [95% CI, −0.01 to 0.03; *P* = .46] in the parsimonious model) or from 2021 (β = −0.01 [95% CI, −0.03 to 0.01; *P* = .30] in the fully adjusted model; β = −0.004 [95% CI, −0.02 to 0.02; *P* = .66] in the parsimonious model).

**Conclusions and Relevance:**

Results of this study indicated that research and development investments did not explain the variation in list prices for the 60 drugs in this sample. Drug companies should make further data available to support their claims that high drug prices are needed to recover research and development investments, if they are to continue to use this argument to justify high prices.

## Key Points

**Question:**

Is there an association between how much drug companies spend on the research and development of new drugs and how much they charge for these drugs?

**Findings:**

In this cross-sectional study of 60 new therapeutic agents approved by the US Food and Drug Administration from 2009 to 2018, there was no association between estimated research and development investments and treatment costs based on list prices at the launch of the product or based on net prices a year after launch.

**Meaning:**

Findings of this study suggest that variation in drug prices could not be explained by research and development investments; drug companies should make further data available if they want to use this argument to justify high prices.

## Teaser

This cross-sectional study assesses the association of research and development investments based on data released by drug companies with list and net prices for prescription drugs approved by the US Food and Drug Administration from 2009 to 2018.

## Introduction

The US has the highest prices in the world for new medicines, and in recent years the prices of many therapeutic agents have increased at rates far exceeding the rate of inflation.^1,2^ For instance, from 2008 to 2016, the list prices of brand-name oral medications increased by an average of 9% each year, and list prices of injectable agents increased by an average of 15% each year.^1^ Drug prices are a major public concern in the US, and there is strong, bipartisan political support for reform. A 2021 survey found that more than 80% of US adults favored allowing Medicare to negotiate drug prices,^3^ something Medicare will be allowed to do for certain drugs starting in 2026 following the recent passage of the Inflation Reduction Act.

Over the past decade, legislators in Congress have introduced numerous proposals aimed at putting downward pressure on drug prices.^4^ Drug companies and their trade groups have opposed many of these reforms by arguing that high drug prices are needed to recover research and development investments. Most debates around drug price regulations have centered on how to strike the right balance between lower drug prices and greater incentives for innovation, yet no study has investigated whether there is an association between how much drug companies invest in research and development to develop new drugs and how much they charge for these drugs. If high research and development costs justified high drug prices, then an association between these 2 measures would be expected. The aim of this cross-sectional study was to examine the association between treatment costs and research and development investments for new therapeutic agents approved by the US Food and Drug Administration (FDA) from 2009 to 2018.

## Methods

Because no data were collected from human participants, this study was deemed exempt from institutional review board approval in accordance with the US Department of Health and Human Services’s regulations for the protection of human subjects in research and the ethics policies and procedures at our institutions. We followed the Strengthening the Reporting of Observational Studies in Epidemiology (STROBE) reporting guideline.^5^

### Data Sources 

We obtained from a previous study^6^ data on research and development investments for 63 new drugs approved by the FDA from January 1, 2009, to December 31, 2018. These products were the only ones for which research and development data were publicly available over this period, because companies generally do not reveal how much is spent on developing individual drugs. These products accounted for approximately one-fifth of all drugs authorized by the FDA over the study period (17.7% [63 of 355 drugs]).^6^

The method used to estimate the amount spent by drug companies to bring each drug to market has been previously described.^6^ In brief, information on this amount was obtained from investor reports published by the drug manufacturers. Phase-specific clinical trial success rates were then used to estimate the amount spent on failed trials for other drug candidates. A cost of capital rate of 10.5% per year was applied to reflect the required rate of return for investors. In this way, the estimates accounted for not only the amount spent on the development of the products in question, but also for the probability of failure in the drug industry and the costs associated with acquiring funds from investors.^6^

We obtained data on list and net prices from SSR Health for these products.^7^ We excluded 3 drugs: (1) lorcaserin (Belviq), because the product was withdrawn from the US market in 2020 over safety concerns; (2) deoxycholic acid (Kybella), because the SSR Health database had no pricing data for this product (a cosmetic drug that is unlikely to be covered by insurance); and (3) omadacycline (Nuzyra), because 2 different formulations of this product were initially approved (oral tablet and powder for injection) and were priced differently.

### Estimation of Standardized Treatment Costs

The 60 products in the final sample of this study were grouped into 3 treatment categories: (1) acute, defined as drugs with an expected duration of use of less than 1 year (eg, treatments for infections or for use in emergency situations); (2) chronic, defined as drugs with an expected duration of use of 1 year or more (eg, treatments for long-lasting conditions); and (3) cycle, defined as drugs with a cyclical dose (eg, treatments for cancer). For acute drugs, we calculated the number of units required for the maximum length of treatment recommended by the FDA. For both chronic and cycle drugs, we calculated the number of units required to provide an annual course of treatment.

To calculate the number of units needed for a standardized treatment, we assumed a standard dose according to the FDA-approved package label of each drug. For drugs with a dose based on the weight of patients, 90 kg was used to estimate the doses for drugs indicated for adults and 45 kg for drugs primarily used in pediatric populations. A standard height of 1.75 m was used to calculate drugs with a dose based on height.

To calculate total treatment costs, we multiplied the total number of drug packages necessary for a standardized treatment by the price per package. For drugs supplied in nonoral formulations (eg, powder for injection), the total cost of treatment was calculated after the total number of packages was rounded to the nearest whole package size.

Total treatment costs were calculated by multiplying the number of units by the price per unit, with costs estimated separately using list and net prices. Treatment costs accounted for only the cost of the drug product. Costs related to the administration of the therapy, or any other treatment-related costs, were not included. Net prices were available for 20 products 1 year after launch and for 21 products in 2021 in the SSR Health database.

### Covariates

For each of the 60 products, we recorded (1) whether a product was first in class or next in class, (2) whether it qualified for any expedited regulatory pathway (ie, accelerated approval, breakthrough, fast track, priority review, or orphan), (3) its route of administration (ie, oral, injection, intravenous, or other), (4) its period of market exclusivity, and (5) its clinical benefit. Information on whether a product was first in class or next in class was gathered from publications by FDA officials.^8,9^ Data on route of administration and whether a product qualified for any expedited regulatory pathway were obtained from the Drugs@FDA database.^10^

We used a published method to estimate the period of market exclusivity for each product.^11^ For small-molecule drugs (ie, drugs with type 1 new drug applications), we estimated the length of exclusivity in 4 steps. First, we checked whether the US patent term for the product was extended under 35 USC §156.^12^ If the product was not granted an extension, we recorded the first patent expiry date in the FDA Orange Book. Second, we identified the statutory exclusivity period for each product. All products in the sample were eligible for a 5-year statutory period of regulatory exclusivity, except products that were granted an orphan designation, which were eligible for a minimum 7-year period of exclusivity. Third, we selected the later date between steps 1 and 2 as the date of loss of exclusivity. Fourth, if the product was granted a 6-month pediatric extension in the FDA database of Pediatric Exclusivity Granted, then 6 months were added to the date from step 3. We followed the same steps for biologics (ie, drugs with biologic license applications), except that we used the Merck Index (instead of the Orange Book) to record the first patent expiry date in step 1, and all biologics were eligible for a 12-year statutory period of regulatory exclusivity in step 2.

We used data published by Haute Autorité de Santé, the French national health technology assessment agency, to assess whether each drug provided added benefit over existing treatment options at the time of launch. We used the assessment of the French agency because no equivalent assessment was available from a US organization. The agency gives all new products that launch in France a score ranging from 1 (for products that offer a major clinical improvement over the best available therapy) to 5 (for drugs that provide no clinical improvement). Scores were available for 38 of the 60 products in the sample. Some older products had been evaluated by the agency more than once, but we used the first score for all products to ensure consistency.

### Statistical Analysis

We calculated correlation coefficients to test the association between research and development investments and treatment costs expressed in linear (Spearman correlation) and log-transformed (Pearson correlation) terms for the 60 products in the study sample, both at the time of launch and in 2021. Log-adjusted costs were used because costs were not normally distributed. We separately calculated correlation coefficients using the available net price data. All research and development figures and prices were adjusted to 2021 US dollars using the US Consumer Price Index.

Because factors other than research and development investments may alter the prices set by drug companies, we also fitted multivariable regression models. In the primary analysis, we ran linear regression models with log-transformed treatment costs as the dependent variable. To select independent variables, we tested for associations between product characteristics and standardized treatment costs using univariate regressions. For the characteristics that were associated with costs, we conducted independence tests to identify characteristics that were highly correlated. We then built 2 models. The first was a fully adjusted model that controlled for all independent variables that were significantly associated with treatment costs. The second was a parsimonious model in which we excluded variables that were highly correlated to prevent multicollinearity. We ran each model using log-transformed treatment costs at launch and log-transformed treatment costs from 2021.

In the secondary analysis, we repeated the same process using generalized linear models with γ distributions and log links. We again ran univariate regressions to identify the variables associated with treatment costs, and then we conducted independence tests to identify correlated variables. We built a fully adjusted model and a parsimonious model. Each model was run using treatment costs at launch and in 2021.

All statistical tests were 2-sided, with *P* < .05 considered to be statistically significant. All statistical calculations and plots were performed with R, version 4.2 (R Foundation for Statistical Computing).

## Results

A total of 60 new FDA-approved therapeutic agents were analyzed. No correlation was observed between estimated research and development investments and log-adjusted treatment costs based on list prices at launch (*R* = −0.02 and *R*^2^ = 0.0005; *P* = .87) or net prices 1 year after launch (*R* = 0.08 and *R*^2^ = 0.007; *P* = .73) (Figure). No correlations were found for treatment costs based on list prices at launch (*R* = 0.02 and *R*^2^ = 0.0004; *P* = .88) and list prices from 2021 (*R* = −0.04 and *R*^2^ = 0.002; *P* = .73). The results were nonsignificant for all combinations of years of price data (launch vs 2021), net price vs list price, and treatment costs vs log-adjusted treatment costs.

**Figure.**
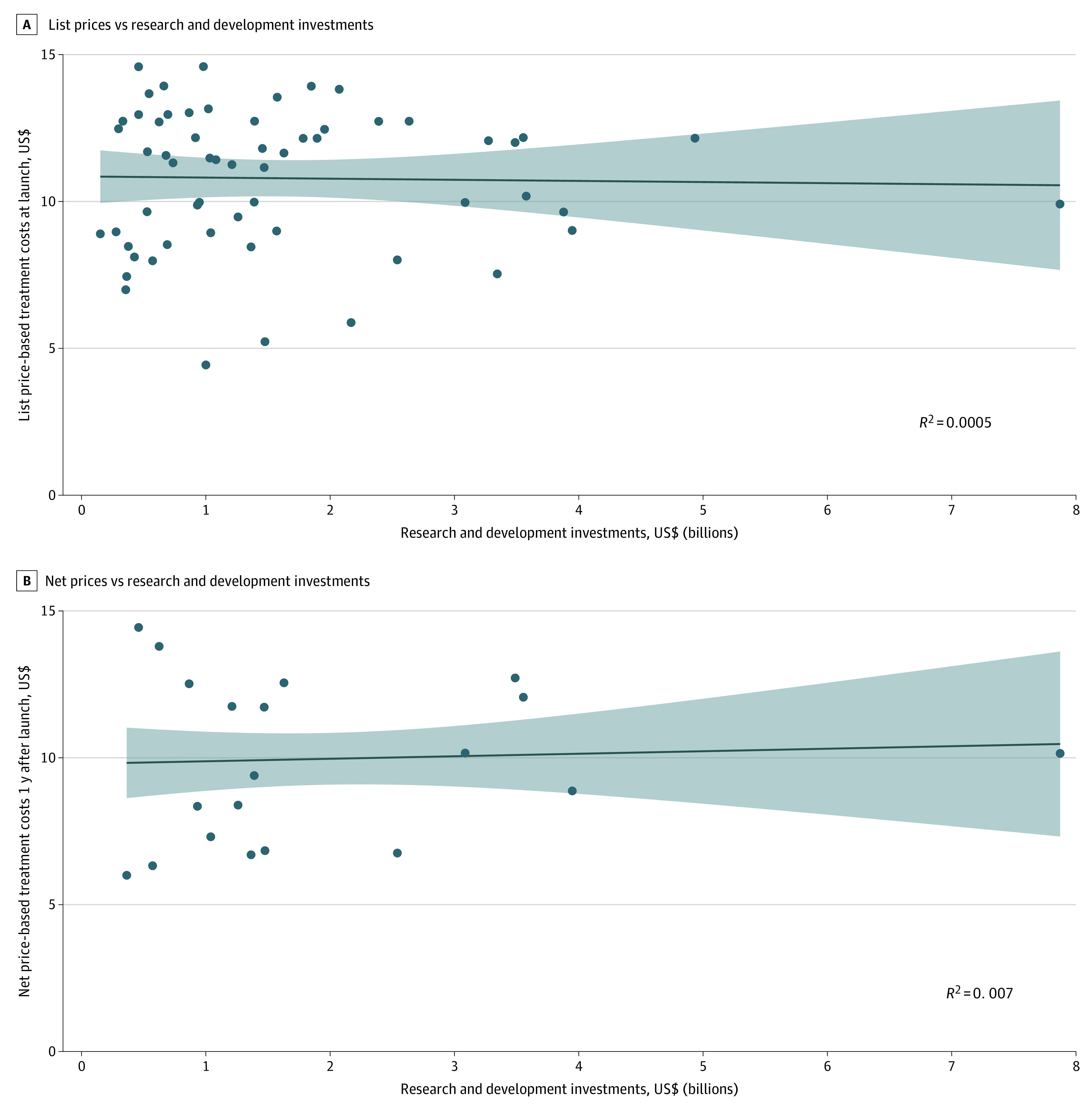
Research and Development Investments vs Log-Adjusted Treatment Costs at Launch Based on List Prices and Net Prices A, List prices were available for 60 products (circles) in the SSR Health database. B, Net prices were available for 20 products (circles) and were only available starting 1 year after product launch. All costs and research and development figures were reported in 2021 US dollars. The plots include lines of best fit, with shaded areas representing the 95% CIs.

The product characteristics associated with log-adjusted treatment costs were type of agent (ie, acute, cycle, or chronic drug), whether a product was first in class or next in class, orphan status, priority review designation, breakthrough therapy designation, and whether the product qualified for accelerated approval (Table 1). These variables were included in the fully adjusted model. Because first in class, priority review designation, and accelerated approval were all correlated with orphan status (Table 2), these variables were excluded in the parsimonious model. eTable 1 and eTable 2 in the Supplement show these results for the secondary analysis based on treatment costs expressed on a linear scale.

**Table 1.** Univariate Associations Between Product Characteristics and Log-Adjusted Treatment Costs^a^

Product category	Log-adjusted treatment costs at launch (based on list prices)		Log-adjusted treatment costs from 2021 (based on list prices)	
	Estimate	*P* value^b^	Estimate	*P* value^b^
Orphan	3.45	<.001	3.40	<.001
First in class	1.40	.02	1.38	.02
Accelerated approval	1.90	.01	1.92	.01
Fast track	0.78	.21	0.60	.32
Breakthrough therapy	1.63	.02	1.43	.04
Priority review	2.09	.001	1.86	.003
Route (oral)	0.05	.93	0.28	.64
Duration of exclusivity	0.20	.07	0.15	.17
Treatment category (reference category: chronic)	NA	<.001	NA	<.001
Acute	−3.93	NA	−3.87	NA
Cycle	1.48	NA	1.43	NA
Clinical benefit (reference category: 5)	NA	.17	NA	.11
2	0.75	NA	0.57	NA
3	1.32	NA	1.44	NA
4	1.76	NA	1.82	NA

Abbreviation: NA, not applicable.

^a^ Associations were estimated using linear regression models. Results were based on data for all 60 products in the sample, except the results for clinical benefit, which were based on 38 products with available data. No drug was given a clinical benefit score of 1.

^b^ *P* values were derived from type 3 tests.

**Table 2.** Independence Tests Among Selected Product Characteristics

Product category	*P* value^a^				
	First in class	Accelerated approval	Breakthrough therapy	Priority review	Treatment category
Orphan	.001	<.001	.13	.002	.14
First in class	NA	.76	.76	.27	.25
Accelerated approval	NA	NA	.72	.03	.29
Breakthrough therapy	NA	NA	NA	.003	.18
Priority review	NA	NA	NA	NA	.91

Abbreviation: NA, not applicable.

^a^ *P* values were derived from 2-sided Fisher exact tests. Results were based on data for all 60 products in the sample.

The linear regression models showed no association between estimated research and development investments and log-adjusted treatment costs at launch (β = 0.002 [95% CI, −0.02 to 0.02; *P* = .84] in the fully adjusted model; β = 0.01 [95% CI, −0.01 to 0.03; *P* = .46] in the parsimonious model) (Table 3) or from 2021 (β = −0.01 [95% CI, −0.03 to 0.01; *P* = .30] in the fully adjusted model; β = −0.004 [95% CI, −0.02 to 0.02; *P* = .66] in the parsimonious model) (Table 4). The parsimonious model identified orphan status and treatment category as the only factors associated with log-adjusted treatment costs at launch.

**Table 3.** Results of Multivariable Linear Regression Models Using Log-Adjusted Treatment Costs at Launch, Based on List Prices

Variable	Fully adjusted model^a^		Parsimonious model^a^	
	β (95% CI)	*P* value	β (95% CI)	*P* value
Intercept	9.13 (8.47 to 9.80)	<.001	9.41 (8.74 to 10.07)	<.001
R&D investment per $100 million	0.002 (−0.02 to 0.02)	.84	0.01 (−0.01 to 0.03)	.46
Orphan	2.72 (1.85 to 3.58)	<.001	2.84 (2.20 to 3.47)	<.001
Acute treatment (reference category: chronic)	−3.13 (−3.98 to −2.28)	<.001	−2.91 (−3.79 to −2.03)	<.001
Cycle treatment (reference category: chronic)	1.23 (0.41 to 2.06)	.004	1.15 (0.31 to 1.98)	.01
Breakthrough therapy	−0.16 (−0.89 to 0.58)	.67	0.20 (−0.53 to 0.93)	.59
First in class	0.06 (−0.61 to 0.73)	.86	NA	NA
Accelerated approval	−0.65 (−1.46 to 0.15)	.11	NA	NA
Priority review	0.97 (0.24 to 1.69)	.01	NA	NA

Abbreviations: NA, not applicable; R&D, research and development.

^a^ The outcome variable in both models was based on list prices (n = 60), with treatment costs measured at launch and expressed using logarithmic scales.

**Table 4.** Results of Multivariable Linear Regression Models Using Log-Adjusted Treatment Costs From 2021, Based on List Prices

Variable	Fully adjusted model^a^		Parsimonious model^a^	
	β (95% CI)	*P* value	β (95% CI)	*P* value
Intercept	9.68 (9.06 to 10.30)	<.001	9.92 (9.31 to 10.54)	<.001
R&D investment per $100 million	−0.01 (−0.03 to 0.01)	.30	−0.004 (−0.02 to 0.02)	.66
Orphan	2.55 (1.75 to 3.36)	<.001	2.73 (2.14 to 3.31)	<.001
Acute treatment (reference category: chronic)	−3.17 (−3.97 to −2.38)	<.001	−2.98 (−3.79 to −2.17)	<.001
Cycle treatment (reference category: chronic)	1.18 (0.41 to 1.95)	.003	1.09 (0.32 to 1.86)	.01
Breakthrough therapy	−0.23 (−0.91 to 0.46)	.51	0.07 (−0.60 to 0.74)	.83
First in class	0.13 (−0.49 to 0.76)	.67	NA	NA
Accelerated approval	−0.50 (−1.26 to 0.25)	.19	NA	NA
Priority review	0.83 (0.15 to 1.51)	.02	NA	NA

Abbreviations: NA, not applicable; R&D, research and development.

^a^ The outcome variable in both models was based on list prices (n = 60), with treatment costs measured from 2021 and expressed using logarithmic scales.

The generalized linear models showed no association between estimated research and development investments and treatment costs at launch (β = −0.02 [95% CI, −0.05 to 0.003; *P* = .08] in the fully adjusted model; β = −0.005 [95% CI, −0.03 to 0.02; *P* = .71] in the parsimonious model) (eTable 3 in the Supplement). A small inverse association was found between estimated research and development investments and treatment costs from 2021 in the fully adjusted model (β = −0.03; 95% CI, −0.05 to −0.01; *P* = .01) but not in the parsimonious model (β = −0.01; 95% CI, −0.03 to 0.01; *P* = .19) (eTable 4 in the Supplement).

## Discussion

To our knowledge, this study was the first to quantitatively explore whether research and development investments were associated with drug prices in the US. Research and development investments for the 60 new drugs examined did not explain variation in list prices, providing little support for arguments by pharmaceutical manufacturers that high drug prices are justified by high research and development costs. Although this empirical evaluation was limited by the number of drugs with available data, the findings were robust to the choice of covariates and the years of the price data (launch vs 2021). A small inverse association between research and development investments and treatment costs from 2021 was observed when generalized linear models were fitted, but this association disappeared in the parsimonious model in which correlated covariates were excluded. No association was found between research and development investments and treatment costs that were estimated using net prices for the subset of products for which net pricing data were available.

The lack of association between research and development investments and list prices of drugs was not unexpected, given that pharmaceutical firms aim to maximize profits based on consumers’ willingness to pay. This study offers empirical evidence that, in the US, drug companies charge what the market will bear. The lack of association between research and development investments and list prices of drugs is, however, of major policy relevance because drug companies and their trade associations often claim that high drug prices are needed to recover research and development investments. If research and development costs justified drug prices, an association between the 2 variables would be found.

Although there was no association between research and development investments and the prices of medicines, this study did not address whether lower industry revenues would be associated with fewer treatments. Simulation studies have suggested that lower revenues in the drug industry may result in decreased research and development investment and, subsequently, fewer new treatments.^13,14,15,16^ However, the clinical significance of such an outcome remains uncertain because it is unclear whether any drugs forgone would represent valuable improvements over existing therapies. Even if they did represent improvements, the detrimental implications of delays in innovation might be offset by the benefits associated with improved access to existing treatments given that an estimated 29% of US patients currently report forgoing medications because of costs.^17^

In theory, many factors could be associated with prices charged by drug companies for their products. These factors include the therapeutic value of a product, aggregate demand for a drug (which is largely associated with disease prevalence), duration of market exclusivity, price sensitivity of demand (as companies may charge higher prices if they believe consumers are unlikely to discontinue therapy), competitiveness of the market (the availability of therapeutic substitutes could put downward pressure on drug prices, although the evidence is mixed on whether this happens in practice^18,19^), and the portfolio of the company (with larger firms selling many products and potentially adopting different pricing strategies vs smaller firms with few products on the market). Many of these factors were accounted for, at least partially, through variables in the present study, such as duration of exclusivity, orphan status (proxy for aggregate demand), clinical benefit (proxy for therapeutic value), and whether a product was first in class or next in class (proxy for degree of competition). We observed that first-in-class and orphan drugs were associated with higher costs at launch. Other factors, such as clinical benefit and duration of exclusivity, had no consistent association with how much drug companies charged for the therapeutic agents in the sample. Although there is growing consensus that the prices of new drugs should be aligned with the value the new products deliver, we found no association between the clinical benefit of a new product and prices. This finding is in line with results from a previous study of cancer therapies.^20^

### Limitations

This study has several limitations. First, the sample size was small, owing primarily to the lack of publicly available data on research and development investments. Second, the primary analysis focused on list prices, which did not reflect discounts, whereas the secondary analysis focused on net prices. Net pricing data were available for a small number of products because SSR Health compiles information for only top-selling products manufactured by publicly traded companies. Third, we were unable to capture all of the factors associated with the prices charged by drug companies for their products, such as the number of competitors already on the market. Fourth, it was difficult to isolate research and development costs for any one product because investments may reflect spillovers in knowledge and resources from earlier efforts. It was also difficult to accurately track all of the preclinical investments because drug companies often do not start reporting costs for individual agents during the early stages of preclinical research (because they may not yet know which drug candidates they will pursue). Both issues affected the study from which we obtained the data on research and development investments.^6^

## Conclusions

In this cross-sectional study, research and development investments for 60 new agents approved by the FDA from 2009 to 2018 did not explain the variation in list prices. Drug companies should supply further data to support claims that high drug prices are needed to recover research and development investments, if this argument will continue to be used to justify high prices.

## Notes

Supplement. **eTable 1.** Univariate Associations Between Product Characteristics and Treatment Costs **eTable 2.** Independence Tests Among Selected Product Characteristics for the Generalized Linear Regression Models **eTable 3.** Results of Generalized Linear Regression Models Using Treatment Costs at Launch (Based on List Prices) **eTable 4.** Results of Generalized Linear Regression Models Using Treatment Costs From 2021 (Based on List Prices)
