# Supplementary material for: Association of Research and Development Investments With Treatment Costs for New Drugs Approved From 2009 to 2018
Source: JAMA Netw Open. 2022 Sep 26;5(9):e2218623. doi: 10.1001/jamanetworkopen.2022.18623 (PMC9513642; doi:10.1001/jamanetworkopen.2022.18623)
Supplement: Supplement. — eTable 1. Univariate Associations Between Product Characteristics and Treatment Costs eTable 2. Independence Tests Among Selected Product Characteristics for the Generalized Linear Regression Models eTable 3. Results of Generalized Linear Regression Models Using Treatment Costs at Launch (Based on List Prices) eTable 4. Results of Generalized Linear Regression Models Using Treatment Costs From 2021 (Based on List Prices) [file jamanetwopen-e2218623-s001.pdf]

Supplemental Online Content

Wouters OJ, Berenbrok LA, He M, Li Y, Hernandez I. Association of research and development investments with treatment costs for new drugs approved from 2009 to 2018. *JAMA Netw Open*. 2022;5(6):e2218623.
doi:10.1001/jamanetworkopen.2022.18623

eTable 1. Univariate Associations Between Product Characteristics and Treatment Costs

eTable 2. Independence Tests Among Selected Product Characteristics for the Generalized Linear Regression Models

eTable 3. Results of Generalized Linear Regression Models Using Treatment Costs at Launch (Based on List Prices)

eTable 4. Results of Generalized Linear Regression Models Using Treatment Costs From 2021 (Based on List Prices)

This supplemental material has been provided by the authors to give readers additional information about their work.

© 2022 Wouters OJ et al. *JAMA Network Open.*

eTable 1. Univariate Associations Between Product Characteristics and Treatment Costs

Product category	Treatment costs at launch (based on list prices)		Treatment costs from 2021 (based on list prices)	
	**Estimate**	**P value**	**Estimate**	**P value**
Orphan	2.67	<.001*	2.71	<.001*
First in class	1.03	.01*	0.87	.047*
Accelerated approval	0.53	.34	0.65	.22
Fast track	0.39	.40	0.28	.54
Breakthrough therapy	0.44	.40	0.34	.51
Priority review	1.29	.01*	1.08	.03*
Route (oral)	-1.03	.005*	-0.94	.008*
Duration of exclusivity	0.10	.23	0.09	.25
Treatment category (reference category: chronic)	-	<.001*	-	<.001*
Acute	-4.10	-	-3.77	-
Cycle	0.92	-	1.07	-
Clinical benefit (reference category: 5)	-	.89	-	.88
2	-1.37	-	-1.49	-
3	0.04	-	0.15	-
4	-0.22	-	-0.06	-

Notes: Associations were estimated using generalized linear models with gamma distributions and log links. Results significant at α level of .05, as indicated by an asterisk (*). Results based on data for all 60 products in the sample, except the results for clinical benefit which were based on 38 products with available data; no drug was given a clinical benefit score of 1. P values were derived from type 3 tests.

© 2022 Wouters OJ et al. *JAMA Network Open.*

eTable 2. Independence Tests Among Selected Product Characteristics for the Generalized Linear Regression Models

**Product category**	First in class	Accelerated approval	Breakthrough therapy	Priority review	Route (oral)	Treatment category
Orphan	.001*	<.001*	.13	.002*	.61	.14
First in class		.76	.76	.27	.44	.25
Accelerated approval			.72	.03*	.13	.29
Breakthrough therapy				.003*	.36	.18
Priority review					.18	.91
Route (oral)						.02*

Notes: Results significant at α level of .05, as indicated by an asterisk (*). P values from two-sided Fisher's exact tests. Results based on data for all 60 products in the sample.

© 2022 Wouters OJ et al. *JAMA Network Open.*

eTable 3. Results of Generalized Linear Regression Models Using Treatment Costs at Launch (Based on List Prices)

	Fully adjusted model		Parsimonious model	
**Variable**	**β (95% CI)**	**P value**	**β (95% CI)**	**P value**
Intercept	10.44 (9.58 to 11.30)	<.001	10.49 (9.72 to 11.26)	<.001
R&D investment (per $100 million)	-0.02 (-0.05 to 0.003)	.08	-0.005 (-0.03 to 0.02)	.71
Orphan	1.82 (1.09 to 2.55)	<.001	2.43 (1.74 to 3.12)	<.001
Acute treatment (reference category: chronic)	-3.41 (-4.31 to -2.51)	<.001	-3.08 (-4.04 to -2.13)	<.001
Cycle treatment (reference category: chronic)	0.64 (-0.23 to 1.52)	.15	1.04 (0.15 to 1.93)	.02
Route (oral)	-0.74 (-1.42 to -0.06)	.03	-	-
First in class	0.30 (-0.37 to 0.97)	.38	-	-
Priority review	1.21 (0.56 to 1.86)	<.001	-	-

Abbreviations: R&D, research and development.

Notes: The generalized linear models used gamma distributions and log links, with results significant at α level of .05. The outcome variable in both models was standardized treatment costs calculated using list prices at launch (n=60).

© 2022 Wouters OJ et al. *JAMA Network Open.*

eTable 4. Results of Generalized Linear Regression Models Using Treatment Costs From 2021 (Based on List Prices)

	Fully adjusted model		Parsimonious model	
**Variable**	**β (95% CI)**	**P value**	**β (95% CI)**	**P value**
Intercept	10.65 (9.90 to 11.39)	<.001	10.78 (10.13 to 11.42)	<.001
R&D investment (per $100 million)	-0.03 (-0.05 to -0.01)	.01	-0.01 (-0.03 to 0.01)	.19
Orphan	1.86 (1.21 to 2.50)	<.001	2.36 (1.78 to 2.94)	<.001
Acute treatment (reference category: chronic)	-3.41 (-4.20 to -2.61)	<.001	-3.12 (-3.95 to -2.28)	<.001
Cycle treatment (reference category: chronic)	0.78 (-0.001 to 1.57)	.05	1.05 (0.28 to 1.82)	.01
Route (oral)	-0.48 (-1.08 to 0.12)	.11	-	-
First in class	0.33 (-0.27 to 0.94)	.28	-	-
Priority review	0.99 (0.42 to 1.55)	.001	-	-

Abbreviations: R&D, research and development.

Notes: The generalized linear models used gamma distributions and log links, with results significant at α level of .05. The outcome variable in both models was standardized treatment costs calculated using list prices in 2021 (n=60).

© 2022 Wouters OJ et al. *JAMA Network Open.*
